# The seroprevalence of anti-*Histoplasma capsulatum* IgG antibody among pulmonary tuberculosis patients in seven referral tuberculosis hospitals in Indonesia

**DOI:** 10.1371/journal.pntd.0011575

**Published:** 2023-09-20

**Authors:** Tutik Kusmiati, Erlina Burhan, Retna Indah Sugiyono, Dona Arlinda, Adhella Menur Naysilla, Banteng Hanang Wibisono, Riat El Khair, Ni Wayan Candrawati, Bintang Yinke Magdalena Sinaga, Irawaty Djaharrudin, Dewi Lokida, Herman Kosasih, Nugroho Harry Susanto, Deni Pepy Butar Butar, Robiatul Adawiyah, Ayu Eka Fatril, Muhammad Karyana, David W. Denning, Retno Wahyuningsih

**Affiliations:** 1 Department of Pulmonology, Soetomo Hospital, Surabaya, Indonesia; 2 Department of Pulmonology, Persahabatan Hospital, Jakarta, Indonesia; 3 Indonesia Clinical Research Center (INA-CRC), Health Policy Agency, Ministry of Health, Jakarta, Indonesia; 4 Department of Internal Medicine, Kariadi Hospital, Semarang, Indonesia; 5 Department of Clinical Pathology and Laboratory Medicine, Faculty of Medicine, Public Health and Nursing, Universitas Gadjah Mada, Sardjito Hospital, Yogyakarta, Indonesia; 6 Department of Pulmonology, Faculty of Medicine, Universitas Udayana, Prof. IGNG. Ngoerah General Hospital, Bali, Indonesia; 7 Department of Pulmonology and Respiratory Medicine, Adam Malik Hospital, Medan, Indonesia; 8 Department of Pulmonology, Faculty of Medicine, Universitas Hasanuddin, Wahidin Sudirohussodo Hospital, Makassar, Indonesia; 9 Department of Clinical Pathology, Tangerang District Hospital, Banten, Indonesia; 10 Department of Parasitology, Universitas Indonesia, Jakarta, Indonesia; 11 Manchester Fungal Infection Group, The University of Manchester, Manchester Academic Health Science Centre, Manchester, United Kingdom; 12 Department of Parasitology, Faculty of Medicine, Universitas Kristen Indonesia, Jakarta, Indonesia; Albert Einstein College of Medicine, UNITED STATES

## Abstract

**Background:**

*Histoplasma capsulatum* exposure is rarely suspected in Indonesia. Pulmonary histoplasmosis can occur simultaneously with pulmonary tuberculosis (TB) or as an alternative diagnosis in clinically-diagnosed TB patients with no microbiological evidence of TB. This study aimed to determine the seroprevalence of anti-*H*. *capsulatum* IgG antibody among pulmonary TB patients.

**Methodology:**

This was a sub-study of 306 participants from a prospective cohort pulmonary TB study conducted at seven TB referral hospitals in Indonesia. The study population was presumptive pulmonary TB adult patients who underwent microbiological TB examinations and were categorized as drug-sensitive (DS), drug-resistant (DR), and clinically-diagnosed TB. Anti-*H*. *capsulatum* IgG antibody levels at baseline were measured using MVista Histoplasma Ab enzyme immunoassays. Data were summarized using descriptive statistics. Bivariate and multivariate logistic regression analysis were performed to assess factors associated with anti-*H*. *capsulatum* IgG antibody positive result.

**Results:**

12.7% (39/306) of pulmonary TB patients were positive for anti-*H*. *capsulatum* IgG antibodies (DR-TB patients (15.9%, 18/114), DS-TB (13.0%, 15/115), and clinically-diagnosed TB (7.8%, 6/77)). The median unit value of anti-*H*. *capsulatum* IgG antibody for all positive samples was 15.7 (IQR 10.2–28.9) EU. This median unit value was higher in clinically-diagnosed TB patients compared to DS-TB or DR-TB patients (38.1 (IQR 25.6–46.6) EU, 19.7 (IQR 12.3–28.9) EU, and 10.9 (IQR 9.2–15.4), respectively). There were 10 patients (3.3%) with anti-*H*. *capsulatum* IgG antibody levels above 30 EU. Factors associated with the anti-*H*. *capsulatum* IgG antibody positive result were malignancies (OR 4.88, 95% CI 1.09–21.69, p = 0.037) and cavitary lesions (OR 2.27, 95% CI 1.09–4.70, p = 0.028).

**Conclusions:**

Our results provide evidence of exposure to *H*. *capsulatum* among pulmonary TB patients in Indonesia. Further studies are needed to provide a comprehensive picture of this fungal disease in other populations and regions to enhance awareness among clinicians and public health officials.

## Author summary

*Histoplasma capsulatum* exposure is rarely suspected in Indonesia, even though this fungal presence in the country has been reported since the 1930s. Pulmonary manifestations of histoplasmosis, especially chronic pulmonary histoplasmosis (CPH), resemble pulmonary tuberculosis (TB). It may be misdiagnosed as or be a co-infection with pulmonary TB. Since Indonesia is the second-highest TB-burdened country worldwide, it is imperative to capture histoplasmosis data among pulmonary TB patients. Our study provides evidence of *H*. *capsulatum* exposure by measuring anti-*H*. *capsulatum* IgG antibodies in both bacteriologically confirmed and clinically-diagnosed pulmonary TB patients at seven TB referral hospitals in Indonesia. We found 12.7% (39/306) of pulmonary TB patients were positive for anti-*H*. *capsulatum* IgG antibodies. We had a high suspicion of pulmonary histoplasmosis diagnosis in 10 of the 39 patients (six bacteriologically confirmed and four clinically-diagnosed TB) who were immunocompromised due to high IgG assay values (>30 EU). The utilization of other modalities, such as the *Histoplasma* antigen test, may support a histoplasmosis diagnosis. In our study, malignancies and cavitary lesions were associated with anti-*H*. *capsulatum* IgG antibody positive results. Our results bring much-needed preliminary data to guide policymakers regarding fungal infection in pulmonary TB patients and support further research on histoplasmosis in Indonesia.

## Introduction

*Histoplasma capsulatum* is a thermally dimorphic fungus that lives in the environment, particularly in soil and the excrement of birds, bats, and chickens [1]. *H*. *capsulatum* microconidia or small hyphal elements can contaminate the surrounding air and infect human lungs, where it transforms into yeast [2]. The infection results in a wide variety of clinical presentations, including asymptomatic; acute (symptom duration <1 month), sub-acute (1–3 months), or chronic pulmonary (>3 months); and life-threatening disseminated histoplasmosis [3,4]. *Histoplasma spp*. is a World Health Organization (WHO) high-priority fungal pathogen and a cause of invasive mycosis included in the WHO list of neglected tropical diseases (NTDs) [5,6]. The importance of this invasive fungal infection is mainly seen in HIV-positive patients, where it is one of the most common opportunistic infections and causes significant morbidity and mortality [7]. Several immunocompromised conditions aside from HIV infection also increase the likelihood of severe histoplasmosis, including solid organ transplantation, chronic inflammatory diseases, autoimmune diseases, innate immunodeficiencies, inborn T-cell immunodeficiencies, poorly controlled diabetes, and hematologic malignancies [2,3,8–10]. Even though histoplasmosis is self-limited in most immunocompetent patients, large fungal inoculums can cause acute severe pulmonary histoplasmosis. Unrecognized infections may become chronic and reactivate later in life when the individual becomes immunocompromised [11].

High endemicity of histoplasmosis is reported in some areas of North America, Latin America, Africa, and Southeast Asia [12,13]. Although the disease was first identified in Indonesia in 1932, the scarcity of histoplasmosis epidemiological data and general unavailability of diagnostic testing has hindered clinicians from considering it as an etiology of disease, leading to misdiagnosis and improper treatment [14]. Histoplasmosis surveillance using a histoplasmin skin test (HST) was conducted in 1956 (Jakarta, Surabaya, and Kedisan, Bali) and 1997 (Medan), revealing positivity rates of 19.8% and 13.6%, respectively. Though these exceeded estimated global rates of 5–14%, no further follow-up was performed [13]. Published research on histoplasmosis in Indonesia remains limited, with most publications being case reports or case series. It is estimated that the annual incidence rate of histoplasmosis among patients with advanced HIV disease in Indonesia was 1% in 2017 [15]. In one center, 25% (22/88) of sera from patients with pulmonary infection were positive for *Histoplasma* galactomannan antigen [15]. While these limited reports are valuable, the actual disease burden and areas of endemicity remain largely unknown.

The inadequacy of recognizing histoplasmosis in Indonesia is thought to be due to the low clinical suspicion and difficulty distinguishing it from other tropical infectious diseases. Pulmonary manifestations, both clinical and radiological, of histoplasmosis resemble those of TB and other bacterial pneumonias [16]. Misdiagnosis of histoplasmosis in smear-negative cases of pulmonary TB have been reported in several settings [17,18]. Furthermore, TB and pulmonary histoplasmosis can coincide, especially in high TB burden areas such as India, China, Nigeria, and South Africa [17,19]. Histoplasmosis should also be of concern in Indonesia, where the national burden of TB is the second highest globally [20]. In instances where histoplasmosis may be considered in the differential diagnosis, there are limited fungal diagnostic modalities and limited coverage by the national health insurance in Indonesia to confirm clinical suspicion. Culture, the gold standard for histoplasmosis diagnosis, takes days or weeks, requires a biosafety level 3 facility, and has a low sensitivity depending on sample quality [2,17,21]. Non-culture modalities such as antigen or antibody detection and molecular-based testing are faster and much more sensitive. Serum antibody can be helpful in the diagnosis of chronic infection or as evidence of exposure since seropositivity to *H*. *capsulatum* can be detected two weeks after infection and persist for several years [2,17]. Hage et al. reported that antibody testing detected 66.7% of acute, 95.1% of sub-acute, and 83.1% of chronic cases among pulmonary histoplasmosis patients [22].

This study aimed to describe anti-*H*. *capsulatum* IgG antibody seroprevalence among pulmonary TB patients enrolled in a prospective cohort TB study in Indonesia conducted by the Indonesia Research Partnership on Infectious Diseases (INA-RESPOND). The parent study is known as the TB Research of INA-RESPOND on Drug Resistance (TRIPOD) study (NCT02758236) [23].

## Methods

### Ethical approval

This sub-study was approved by the Institutional Review Board (IRB) of Tangerang District Hospital, Tangerang, Indonesia (FWA 00025252) (No. 445/014/KEP/RSUTNG). When signing the informed consent form for the TB Research of INA-RESPOND on Drug Resistance (TRIPOD) study, participants agreed that they were willing to have their specimens stored and used in future studies related to TRIPOD.

### Study design and settings

This was a cross-sectional analysis of specimens and data from TRIPOD study participants. The TRIPOD study was conducted at the following seven TB referral hospitals in Indonesia: Adam Malik Hospital, Medan, North Sumatera; Persahabatan Hospital, East Jakarta, Jakarta; Kariadi Hospital, Semarang, Central Java; Sardjito Hospital, Yogyakarta Special Region; Soetomo Hospital, Surabaya, East Java; Sanglah Hospital, Denpasar, Bali; and Wahidin Sudirohusodo Hospital, Makassar, South Sulawesi with the enrollment period from February 2017 to November 2018. The study population was presumptive pulmonary TB adult patients (with or without a history of anti-TB treatment) attended to the hospital with a cough for at least two weeks and at least one other TB symptom (fever, unexplained weight loss, loss of appetite, hemoptysis, shortness of breath, chest pain, night sweats, or fatigue) and chest X-ray suggestive TB. The TRIPOD study successfully enrolled 490 participants and collected sputum for microbiological TB examinations (acid-fast bacilli (AFB) smear, Xpert MTB/RIF, and *Mycobacterium tuberculosis* (MTB) culture) and blood for laboratory examinations. Participants were terminated from the study if they were not treated as pulmonary TB patients by the clinician. Participants who were treated as pulmonary TB patients, according to microbiological TB and drug susceptibility tests were further categorized as pulmonary TB bacteriologically confirmed, with sub-categorization as drug-sensitive (DS-TB) or drug-resistant (DR-TB), and clinically-diagnosed TB when there was no microbiological evidence of TB [23]. This histoplasmosis study utilized baseline demographic data, clinical data, and repository sera of 306 randomly selected TRIPOD participants who had at least one of the following histoplasmosis risk factors: malnutrition, previous anti-TB treatment history, presence of cavities on chest X-ray, comorbidity of HIV and/ or diabetes, or failed treatment outcome. Serum samples were collected during the first TRIPOD study visit and before anti-TB treatment was initiated. Specimens were stored at -80°C until analysis.

### Laboratory methods

Serum samples were tested for anti-*H*. *capsulatum* IgG antibody using the MVista Histoplasma Antibody Enzyme Immunoassay (EIA) (MiraVista Diagnostics, Indiana, USA) according to the manufacturer’s instructions [24]. The calculation of the test result was determined using a 4-parameter curve to fit the standard curve values to their corresponding optical density (OD) readings. The result was predicted by extrapolation from the curve fit. According to manufacturer instructions, the specimen test result was considered positive if the EIA Unit (EU) value was > 10.0 EU, intermediate if the EU value was 8.0–9.9 EU, and negative if the EU value was < 8.0 EU [24]. Intermediate results were interpreted as positive in this study. An IgG level above 30 EU is considered high based on the report from Richer et al. [25].

### Data and statistical methods

Data were summarized using descriptive statistics, such as frequencies (percentages) and medians (inter-quartile ranges (IQRs)). Differences in baseline characteristics between positive and negative anti-*H*. *capsulatum* IgG antibody samples were evaluated using Pearson Chi-square or Fisher’s exact test, as appropriate. Bivariate logistic regression was used to assess factors associated with anti-*H*. *capsulatum* IgG antibody positive result. All independent variables with p-value <0.25 were included in multivariate logistic regression models and adjusted odds ratios (aORs) with 95% CIs were used to determine the strength of association between the dependent and independent variables. A p-value (two-tailed) <0.05 was considered statistically significant. Statistical Package for Social Science (SPSS) software version 23 (IBM Corporation, Armonk, NY, USA) and GraphPad Prism 9.0 (GraphPad Software, Inc., California, USA) were used for the statistical analysis and graphs. Figures were developed using QGIS 3.32.1 and BioRender (BioRender, Ontario, Canada).

## Results

**Fig 1:**
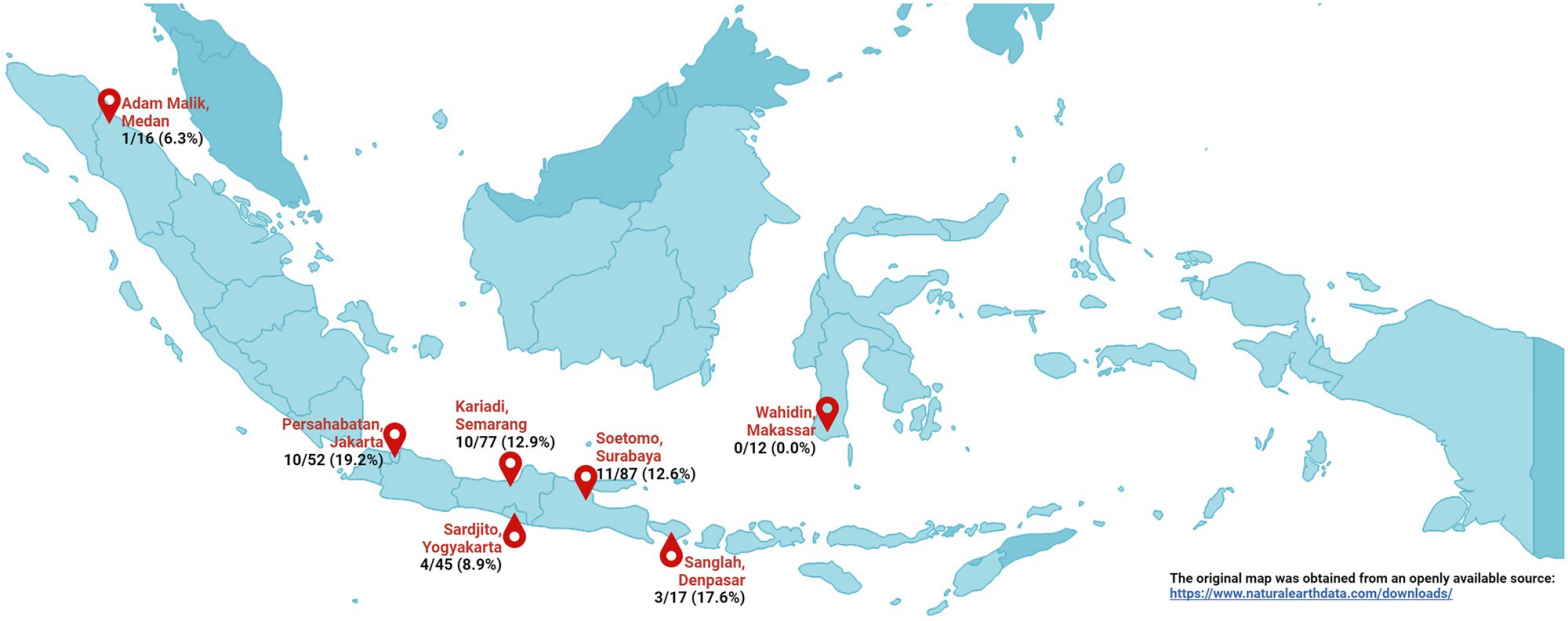
The distribution of anti-*H*. *capsulatum* IgG antibody-positive serum specimens from pulmonary TB patients at seven TB referral hospitals in Indonesia. The original map was obtained from an openly available source https://www.naturalearthdata.com/. The figure was developed with QGIS 3.32.1 and https://biorender.com/.

Serum specimens were *H*. *capsulatum*-positive in 39 of 306 (12.7%) pulmonary TB patients. The seroprevalence was highest among DR-TB patients (15.9%, 18/114), followed by DS-TB patients (13.0%, 15/115) and clinically-diagnosed TB patients (7.8%, 6/77). The three sites with the highest seroprevalence were Persahabatan Hospital, Jakarta (19.2%), Sanglah Hospital, Bali (17.6%), and Kariadi Hospital, Semarang (12.9%). None of the 12 patient specimens from Wahidin Sudirohusodo Hospital, Makassar were positive. The distribution of *H*. *capsulatum*-positive pulmonary TB patients across study sites is shown in Fig 1.

**Fig 2:**
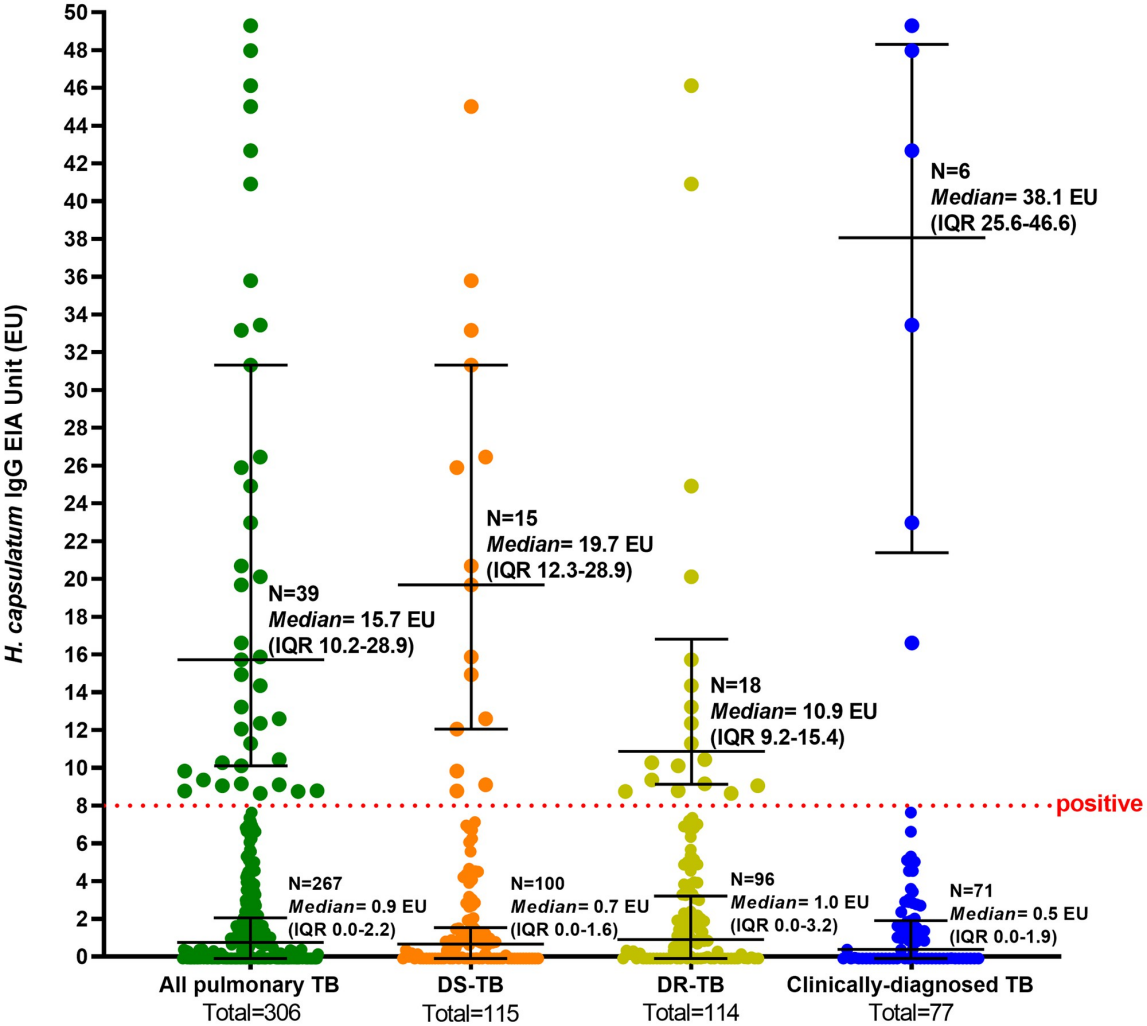
Anti-*H*. *capsulatum* IgG antibody results among DS-TB, DR-TB, and clinically-diagnosed pulmonary TB patients.

The median EU value of positive specimens was 15.7 (IQR 10.2–28.9). Clinically-diagnosed TB patients showed the highest median EU value (38.1 (IQR 25.6–46.6)), followed by DS-TB (19.7 (12.3–28.9)) and DR-TB (10.9 (IQR 9.2–15.4)) (Fig 2).

Table 1 shows the demographic and clinical characteristics of the 39 serum-positive patients according to their TB classification. Patients were mostly male (26/39, 66.7%) and under 60 years old (34/39, 87.2%), with a median age of 45 years old. Over half of the patients were underweight (22/39, 56.4%), and over half had a previous anti-TB treatment history (21/39, 53.8%). TB comorbidities among serum-positive patients included diabetes (16/39, 41.0%), malignancies (3/39, 7.7%), and HIV co-infection (1/39, 2.6%). Among all serum-positive patients, the most frequent symptom was productive cough (38/39, 97.4%), followed by unexplained weight loss (33/39, 84.6%) and fatigue (28/39, 71.2%). 24 patients (61.5%) showed cavities in their chest X-rays, mostly located in upper pulmonary zones (19/24, 79.2%). 9 patients (23.1%) died during the course of their anti-TB treatment, which was higher than the mortality observed in serum-negative patients (38/267, 14.2%) though not statistically significant (p = 0.15) S1 Table. Characteristics of participants based on anti-*H*. *capsulatum* IgG antibody results).

**Table 1:** Characteristics of the anti-*H*. *capsulatum* IgG antibody-positive pulmonary TB patients.

	All (n = 39)	DS-TB (n = 15)	DR-TB (n = 18)	Clinically-diagnosed TB (n = 6)
Gender, n (%)				
Male	26 (66.7)	11 (73.3)	11 (61.1)	4 (66.7)
Female	13 (33.3)	4 (26.7)	7 (38.9)	2 (33.3)
Age, n (%)				
18–59 years old	34 (87.2)	14 (93.3)	17 (94.4)	3 (50.0)
≥ 60 years old	5 (12.8)	1 (6.7)	1 (5.6)	3 (50.0)
BMI (kg/m^2^), n (%)				
< 18.5	22 (56.4)	8 (53.3)	13 (72.2)	1 (16.7)
18.5–24.9	14 (35.9)	7 (46.7)	3 (16.7)	4 (66.7)
> 25	3 (7.7)	0 (0.0)	2 (11.1)	1 (16.7)
Previous anti-TB treatment history, n (%)	21 (53.8)	7 (46.7)	12 (66.7)	2 (33.3)
Comorbidities and coinfections, n (%)				
Anemia	27 (69.2)	12 (80.0)	11 (61.1)	4 (66.7)
Diabetes	16 (41.0)	7 (46.7)	7 (38.9)	2 (33.3)
Malignancies	3 (7.7)	1 (6.7)	0 (0.0)	2 (33.3)
HIV*	1 (2.9)	0 (0.0)	0 (0.0)	1 (20)
Signs and symptoms, n (%)				
Productive cough	38 (97.4)	15 (100)	17 (94.4)	6 (100)
Hemoptysis	13 (33.3)	5 (33.3)	4 (22.2)	4 (66.7)
Fever	24 (61.5)	6 (40.0)	14 (77.8)	4 (66.7)
Unexplained weight loss	33 (84.6)	14 (93.3)	15 (83.3)	4 (66.7)
Loss of appetite	22 (56.4)	8 (53.3)	9 (50.0)	5 (83.3)
Shortness of breath	25 (64.1)	10 (66.7)	14 (77.8)	1 (16.7)
Chest pain	12 (30.8)	6 (40.0)	5 (27.8)	1 (16.7)
Night sweating	23 (58.9)	11 (73.3)	8 (44.4)	4 (66.7)
Fatigue	28 (71.2)	11 (73.3)	15 (83.3)	2 (33.3)
Presence of cavity in chest x-ray, n (%)	24 (61.5)	9 (60.0)	14 (77.8)	1 (16.7)
TB treatment outcome, n (%)				
Cured or completed treatment	18 (46.2)	8 (53.3)	9 (50.0)	1 (16.7)
Lost to follow-up	7 (17.9)	3 (20.0)	4 (22.2)	0 (0.0)
Died	9 (23.1)	2 (13.3)	5 (27.8)	2 (33.3)
Not available	5 (12.8)	2 (13.3)	0 (0.0)	3 (50.0)
Anti-*H*. *capsulatum* IgG (EU), n (%)				
8–30	29 (74.4)	11 (73.3)	16 (88.9)	2 (33.3)
> 30	10 (25.6)	4 (26.7)	2 (11.1)	4 (66.7)

*HIV testing was performed on 35 participants (14 DS-TB, 16 DR-TB, and 5 clinically-diagnosed TB)

Anti-*H*. *capsulatum* IgG antibody levels greater than 30 EU were detected in 10 patients: 4 with DS-TB (45.0, 35.8, 33.2, and 31.3 EU), 2 with DR-TB (46.1 and 40.9 EU), and 4 with clinically diagnosed-TB (49.3, 47.9, 42.7, and 33.4 EU). All of these patients had comorbidities in addition to pulmonary symptoms. The 4 DS-TB patients had poorly controlled diabetes, with HbA1C levels of 13.4%, 11.5%, 12.3%, and 14.4% at baseline, and one of them was also malnourished, with a BMI of 15.6 kg/m^2^. The 2 DR-TB patients were malnourished, with BMIs of 17.3 and 12.9 kg/m^2^. 2 of the clinically-diagnosed TB patients were >60 years and had hematological malignancies (acute lymphocytic leukemia and acute myeloid leukemia), with one of them also having poorly controlled diabetes (HbA1C level of 8%). Both died during anti-TB treatment. The other 2 clinically-diagnosed TB patients included an HIV-positive individual with a baseline CD4+ cell count of 49 cells/L and a >60 years individual with poorly controlled diabetes (HbA1C level of 9.6%). The characteristic details of 39 serum-positive patients (demography, clinical, and laboratory presentations) is provided in S2 Table.

Table 2. presents factors associated with the anti-*H*. *capsulatum* IgG antibody positive result. Cavitary lesions in the lung were significantly associated with the anti-*H*. *capsulatum* IgG antibody positive result as evaluated by bivariate analysis. The multivariate logistic regression analysis included the variables with a p-value of <0.25. Factors independently associated with the anti-*H*. *capsulatum* IgG antibody positive result were malignancies (OR 4.88, 95% CI 1.09–21.69, p = 0.037) and cavitary lesions in the lung (OR 2.27, 95% CI 1.09–4.70, p = 0.028).

**Table 2:** Bivariate and multivariate logistic regression analyses of factors associated with the anti-*H*. *capsulatum* IgG antibody positive result.

	Positive anti-*H*. *capsulatum* IgG No. (%) N = 39	Negative anti-*H*. *capsulatum* IgG No. (%) N = 267	Bivariate analysis		Multivariate analysis	
			OR (95% CI)	p-value	Adjusted OR (95% CI)	p-value
Gender						
Male	26 (66.7)	154 (57.7)	1.47 (0.72–2.98)	0.289		
Female	13 (33.3)	113 (42.3)				
Age (years)						
18–44	18 (46.2%)	147 (55.1%)	*Ref*.			
45–59	16 (41.0%)	92 (34.5%)	1.42 (0.69–2.92)	0.341		
≥60	5 (12.8%)	28 (10.5%)	1.46 (0.50–4.25)	0.490		
Site location with available histoplasmosis surveillance data			1.46 (0.73–2.92)	0.289		
Yes (Bali, Surabaya, Jakarta, and Medan)	25 (64.1)	147 (55.1)				
No (Semarang, Yogyakarta, and Makassar)	14 (35.9)	120 (44.9)				
BMI (kg/m^2^)						
< 18.5 (underweight)	22 (56.4)	133 (49.8)	1.30 (0.66–2.57)	0.442		
≥18.5	17 (43.6)	134 (50.2)				
Previous TB treatment history						
Yes	21 (53.8)	111 (41.6)	**1.64 (0.84–3.22)**	**0.151**	1.73 (0.84–3.55)	0.135
No	18 (46.2)	156 (58.4)				
Anemia						
Yes	27 (69.2)	170 (63.7)	1.28 (0.62–2.65)	0.499		
No	12 (30.8)	97 (36.3)				
Positive HIV (available data in 285 participants)						
Yes	1 (2.6)	16 (5.9)	0.43 (0.06–3.35)	0.420		
No	34 (97.1)	234 (93.6)				
Diabetes						
Yes	16 (41.0)	92 (34.5)	1.32 (0.67–2.63)	0.424		
No	23 (59.0)	175 (65.5)				
Malignancies						
Yes	3 (7.7)	7 (2.6)	**3.09 (0.77–12.51)**	**0.113**	**4.88 (1.09–21.69)**	**0.037**
No	36 (92.3)	260 (97.4)				
Unexplained weight loss						
Yes	33 (84.6)	204 (76.4)	1.69 (0.68–4.24)	0.256		
No	6 (15.4)	63 (23.6)				
Hemoptysis						
Yes	13 (33.3)	85 (31.8)	1.07 (0.52–2.19)	0.851		
No	26 (66.7)	182 (68.2)				
Fever						
Yes	24 (61.5)	164 (61.4)	1.01 (0.50–2.01)	0.989		
No	15 (38.5)	103 (38.6)				
Shortness of breath						
Yes	25 (64.1)	169 (63.3)	1.04 (0.51–2.09)	0.922		
No	14 (35.9)	98 (36.7)				
Chest pain						
Yes	12 (30.8)	114 (42.7)	**0.59 (0.29–1.23)**	**0.161**	0.57 (0.27–1.21)	0.143
No	27 (69.2)	153 (57.3)				
Presence of cavity						
Yes	24 (61.5)	113 (42.3)	**2.18 (1.09–4.34)**	**0.027**	**2.27 (1.09–4.70)**	**0.028**
No	15 (38.5)	154 (57.7)				
TB category						
DS-TB	15 (38.5)	100 (37.5)	*Ref*.			
DR-TB	18 (46.2)	96 (35.9)	1.25 (0.59–2.62)	0.555		
Clinically-diagnosed TB	6 (15.4)	71 (26.6)	0.563 (0.21–1.52)	0.258		

## Discussion

We conducted an initial survey of anti-*H*. *capsulatum* IgG antibody prevalence among pulmonary TB patients participating in the multicenter TRIPOD study. Anti-*H*. *capsulatum* IgG antibody was detected in 12.7% (39/306) of our study population, consistent with previous surveillance reports conducted in several population in Indonesia such as Jakarta, Surabaya, Kedisan, and Medan. In surveillance reports using HST (1953–1954 and 1996–1997), induration of 6 mm or more was identified in 12.5% of 2,542 Jakarta residents (2,275 healthy persons and 267 hospitalized patients), 32% of 282 Surabaya residents, 63.6% of 340 Kedisan residents, and 13.6% of 1,265 Medan residents [13,26,27]. In our study, the proportions of serum-positive pulmonary TB patients with histoplasmosis risk factors from Jakarta, Surabaya, Bali, and Medan were 19.2%, 12.6%, 17.6%, and 6.3%, respectively. Additionally, the proportions of serum-positive patients from Yogyakarta and Semarang were 8.9% and 12.9%, respectively. Though there are numerous differences between our study and the previous results using HST, it is interesting to see that exposure to *H*. *capsulatum* is seemingly common in several areas of Indonesia despite there being little-to-no clinical or epidemiological data on histoplasmosis in the country. Interestingly, no patients from Makassar were serum-positive, though it is difficult to draw meaningful conclusions from this observation given the small sample size of 12. Our study improves upon these previous reports by examining a targeted population in a larger geographical area and using laboratory assays never previously used in Indonesia.

Our study used *H*. *capsulatum*-specific IgG serologic testing with a published sensitivity of 87.5% in clinical and epidemiological cases and a specificity of 95% [25]. The positivity rates from IgG testing can be lower than HST, as reported by Bahr et al. in Burmese refugee samples (1% vs. 4–86%) [28]. HST reflects a cell-mediated immunity response which may remain positive for life in 90% of exposed persons, whereas the humoral immune response producing IgG wanes after several years [28]. However, due to the non-specific polyvalent set of antigens used in HST, cross-reactivity with other endemic fungi such as *Coccidioides spp*. and *Blastomyces dermatitidis* is frequent [2,29]. The anti-*H*. *capsulatum* IgG antibody levels in our 39 positive specimens ranged from 8.64 to 49.3 EU, with a median value of 15.7 EU. This is consistent with the 8.0 to 26 EU results seen with similar assays in other seroprevalence studies from Uganda and Myanmar, Laos, and Somalia [28,30].

Rather than pursuing a sero-surveillance of the general Indonesian population, our study focused on pulmonary TB patients who have overlapping symptoms of histoplasmosis. A recent study from Calabar, Nigeria, concluded the imperativeness of histoplasmosis screening in presumptive pulmonary TB patients [19]. The study reported that 12.7% (27/213) of patients were positive by *Histoplasma* antigen and/or molecular test. Among these, 7 were detected from 94 confirmed TB patients (7.4%), and 20 were detected from 119 unconfirmed TB patients (16.8%) [19]. The detection of anti-*H*. *capsulatum* IgG antibodies indicate prior exposure, asymptomatic infection, acute pulmonary histoplasmosis, or chronic histoplasmosis. Richer et al. reported that 78.8% of 80 definitive pulmonary histoplasmosis cases had IgG assay values >30 EU [25], which we detected in 10 patients from our study. Those patients may have had pulmonary histoplasmosis in addition to TB, though we did not conduct further testing such as respiratory culture, antigen, molecular, IgM in acute samples, or IgG in convalescent samples to completely characterize the serum-positive patients in our study.

Considering our study population and the clinical setting, the presence of anti-*H*. *capsulatum* IgG antibody is most consistent with chronic pulmonary histoplasmosis (CPH). CPH is characterized by low-grade chronic symptoms, tissue destruction, and cavity formation that may lead to progressive pulmonary insufficiency [17]. Cavitation is often acquitted in the upper lobes and is similar to pulmonary TB and chronic pulmonary aspergillosis [17]. The underlying pathogenesis of CPH is chronic inflammation disproportionate to the fungal load, making it challenging to identify [31]. Therefore, multiple assays are required to confirm the CPH diagnosis due to the poor sensitivity of sputum culture and blood antigen testing, the modest sensitivity of bronchoalveolar lavage culture, and the often-low levels of antibody [17]. We were unable to categorize the serum-positive patients in our study as having CPH given that they had the similar clinical presentation of active pulmonary TB. However, given the high exposure of *H*. *capsulatum* that others have documented, as well as the enormous number of chronic pulmonary diseases in Indonesia driven by smoking, asthma, and TB, it is likely that CPH is present in the country and is an underestimated cause of morbidity and mortality [15,18,32].

All 10 patients with high suspicion of pulmonary histoplasmosis based on IgG assay values >30 EU had comorbidities, including 6 patients with diabetes and high HbA1C levels, 2 with malignancies, 3 with malnourishment, and 1 with HIV. In 4 patients where TB was not bacteriologically-confirmed, histoplasmosis is strongly suspected given their risk factors. 3 of those patients were >60 years old, and all 4 patients were immunocompromised due to hematological malignancies, HIV infection, or diabetes [3,33,34]. Patients with hematological malignancies are predisposed to primary or reactivated histoplasmosis due to defects in cell-mediated immunity and chemotherapy side-effects [35]. Peigne et al. described a patient with chronic myeloid leukemia who developed respiratory failure despite anti-TB treatment, later testing positive for histoplasmosis [35]. Bansal et al. reported two fatal acute myeloid leukemia cases with positive *H*. *capsulatum* antigen tests [36]. TB and histoplasmosis are frequently discovered in advanced HIV patients, particularly when CD4+ cell counts are less than 50 cells/μL [37–39]. Patients with CD4+ counts between 0–50 cells/μL had a hazard ratio for developing progressive disseminated histoplasmosis of 47.2 compared to patients with counts >500 cells/μL [39]. In HIV-negative patients, uncontrolled diabetes was the most commonly identified histoplasmosis risk factor in Southeast Asia [13] and may predispose patients to severe disease [40]. Unidentified defects in cell-mediated immunity as a diabetes complication likely result in the patient’s failure to activate macrophage fungicidal capacity against *H*. *capsulatum* [40].

Further investigation of histoplasmosis in the clinically-diagnosed TB population, particularly those with impaired immune systems, is needed. Immunocompromised patients may be unable to mount an antibody response, which reduces the sensitivity of the antibody detection assay used in this study. In a clinical setting, clinicians should combine antibody testing with antigen detection assays to verify a histoplasmosis diagnosis [25]. The importance of diagnosing other pathogens early in non-bacteriologically-confirmed pulmonary TB patients is crucial since empirical anti-TB treatment is common in highly endemic countries like Indonesia. Other non-TB diagnoses are usually considered only after unsuccessful anti-TB treatment. In one center in India, 41.7% of patients were on empirical anti-TB treatment before pulmonary histoplasmosis was diagnosed [41]. A systematic review of 80 fungal infection cases misdiagnosed as TB revealed that 20% of patients had histoplasmosis [18]. Detecting the presence of other pathogens, including *Histoplasma spp*., either as an alternative diagnosis to TB or as a co-infection will benefit patients by enabling accurate early treatment and avoiding unnecessary anti-TB treatment and its consequences, like toxicity and the development of drug resistance. Nevertheless, clinicians frequently prioritize initiating empirical anti-TB treatment immediately to prevent TB transmission, lung destruction, and death.

Pulmonary TB patients are at higher risk of fungal infection due to altered bronchial structure and favorable growth sites in damaged lungs, such as oxygen-rich cavities and necrotic tissues, resulting from current or past TB infection [19,42,43]. Furthermore, pulmonary TB patients may be immunocompromised due to malnutrition, prolonged use of antibiotics, diabetes, and other comorbidities [42–44]. The epidemiology and clinical similarities between pulmonary TB and histoplasmosis, as well as challenges with diagnostic examination for both diseases, confuse clinicians in establishing accurate diagnoses of TB, histoplasmosis, or co-infections of the two. Demographically, working men have more opportunities for exposure due to their work, travel, social networks, and cigarette smoking habits, which reduce lung immunity [43,45]. Clinical presentations of productive cough, weight loss, fatigue, fever, night sweats, persistent cavitation, nodules, and the development of pulmonary fibrosis may overlap [16,17]. Our study found that pulmonary TB patients with malignancies or cavitary lesions in the lung were significantly associated with the anti- *H*. *capsulatum* IgG antibody positive result. Previous studies have described the association between pulmonary cavitation and immunocompromised status with fungal co-infection in pulmonary TB patients [42]. Cavities resemble an ideal culture plate for fungal colonization [42]. Patients with malignancies are exposed to long-term chemotherapy or immunosuppressant that decreases immunity, thus making the patients more susceptible to fungal co-infection [42]. Screening for histoplasmosis may be beneficial in pulmonary TB patients with malignancies or cavitary lesions, both bacteriologically TB-confirmed and clinically-diagnosed TB. However, our findings should be interpreted cautiously because the positive results of the IgG antibody may reflect past exposure. Similar to TB, surviving *H*. *capsulatum* can enter a latent state in macrophages until host immunity wanes, resulting in reactivation [46]. Since histoplasmosis is curable, like TB, understanding this neglected fungal disease is essential for preventing morbidity and mortality.

### Study limitations

Our study is a sub-study of the TRIPOD observational cohort of newly diagnosed and previously treated pulmonary TB patients at seven referral hospitals. The limited scale of this initial survey means that the anti-*H*. *capsulatum* IgG seroprevalence we observed is highly unlikely to represent the seroprevalence among the general TB population or the healthy population of Indonesia. Though the seroprevalence data we collected is of value given the limited data currently available, our use of IgG testing on baseline sera specimens alone prevented us from definitively identifying instances of active histoplasmosis infection or CPH. *Histoplasma* antigen test is feasible in resource-limited settings to support histoplasmosis diagnosis. However, our study was designed to evaluate the seroprevalence of anti-*H*. *capsulatum* IgG antibody; thus, the antigen test was not provided. Also, the antigen test is more useful in establishing disseminated histoplasmosis, which is usually uncommon in pulmonary TB patients. Additionally, we did not collect occupation, travel, or residence information from participants, so we were unable to speculate on the epidemiology of *H*. *capsulatum* exposure and infection in Indonesia. The results of this sub-study provide much needed preliminary data to support further research on histoplasmosis in the country.

## Conclusions

Our results provide evidence of *H*. *capsulatum* exposure in pulmonary TB patients in Indonesia. Pulmonary TB patients, both bacteriologically TB confirmed and clinically-diagnosed TB, with malignancies or cavitary lesions in the lung were associated with the anti- *H*. *capsulatum* IgG antibody positive result in our study. Given that this was a cross-sectional study utilizing serum testing for anti-*H*. *capsulatum* IgG antibodies, we cannot distinguish whether the observed exposures showed acute, chronic, or post-infection states. However, recognizing the occurrence of *H*. *capsulatum* exposure is an important first step in data-limited, high TB burden environments like Indonesia. Patients with pulmonary TB may develop severe histoplasmosis and experience fatal complications that could be avoided if appropriate anti-fungal treatment is initiated. The under-reporting of *H*. *capsulatum* exposure and disease in Indonesia generates an urgent need to increase the awareness and knowledge of clinicians and public health officials.

## Supporting information

S1 Table Characteristics of participants based on anti-*H*. *capsulatum* IgG antibody results. (DOCX)

S2 Table Details of the 39 serum-positive pulmonary TB patients in this study. (DOCX)

S1 Data Anonymized dataset of 306 study participants. (XLSX)
