# Supplementary material for: The seroprevalence of anti-Histoplasma capsulatum IgG antibody among pulmonary tuberculosis patients in seven referral tuberculosis hospitals in Indonesia
Source: PLoS Negl Trop Dis. 2023 Sep 20;17(9):e0011575. doi: 10.1371/journal.pntd.0011575 (PMC10511117; doi:10.1371/journal.pntd.0011575)
Supplement: S1 Table — (DOCX) [file pntd.0011575.s001.docx]

**S1 Table.** Characteristics of participants based on anti-*H. capsulatum* IgG antibody results

| **Characteristic** | **Total**  **No. (%)**  **N = 306** | **Positive anti-*H. capsulatum* IgG**  **No. (%)**  **N = 39** | **Negative anti-*H. capsulatum* IgG**  **No. (%)**  **N = 267** | **p-value** |
| --- | --- | --- | --- | --- |
| Gender |  |  |  |  |
| Male | 180 (58.8) | 26 (66.7) | 154 (57.7) | 0.287 |
| Female | 126 (41.2) | 13 (33.3) | 113 (42.3) |  |
| Age (years) |  |  |  |  |
| 18-44 | 165 (53.9) | 18 (46.2%) | 147 (55.1%) | 0.579 |
| 45-59 | 108 (35.3) | 16 (41.0%) | 92 (34.5%) |  |
| ≥ 60 | 33 (10.8) | 5 (12.8%) | 28 (10.5%) |  |
| Hospital |  |  |  |  |
| Sanglah (Bali) | 17 (5.6) | 3 (7.7) | 14 (5.2) | 0.588 |
| Wahidin (Makasar) | 12 (3.9) | 0 (0.0) | 12 (4.5) |  |
| Kariadi (Semarang) | 77 (25.2) | 10 (25.6) | 67 (25.1) |  |
| Soetomo (Surabaya) | 87 (28.4) | 11 (28.2) | 76 (28.5) |  |
| Sardjito (Yogyakarta) | 45 (14.7) | 4 (10.2) | 41 (15.4) |  |
| Persahabatan (Jakarta) | 52 (17.0) | 10 (25.6) | 42 (15.7) |  |
| Adam Malik (Medan) | 16 (5.2) | 1 (2.6) | 15 (5.6) |  |
| BMI (kg/m^2^) |  |  |  |  |
| < 18.5 (underweight) | 155 (50.7) | 22 (56.4) | 133 (49.8) | 0.441 |
| ≥ 18.5 | 151 (49.3) | 17 (43.6) | 134 (50.2) |  |
| Previous TB treatment history | 132 (43.1) | 21 (53.8) | 111 (41.6) | 0.148 |
| Positive HIV* | 17 (5.6) | 1 (2.6) | 16 (5.9) | 0.704 |
| Anemia | 197 (64.4) | 27 (69.2) | 170 (63.7) | 0.498 |
| Diabetes | 108 (35.3) | 16 (41.0) | 92 (34.5) | 0.423 |
| Malignancies | 10 (3.3) | 3 (7.7) | 7 (2.6) | 0.122 |
| TB category |  |  |  |  |
| DS-TB | 115 (37.6) | 15 (38.5) | 100 (37.5) | 0.265 |
| DR-TB | 114 (37.3) | 18 (46.2) | 96 (35.9) |  |
| Clinically-diagnosed TB | 77 (25.2) | 6 (15.4) | 71 (26.6) |  |
| Signs and symptoms |  |  |  |  |
| Productive cough | 290 (94.8) | 38 (97.4) | 252 (94.4) | 0.703 |
| Dry cough | 124 (40.5) | 16 (41.0) | 108 (40.4) | 0.945 |
| Coughs worsen at night | 146 (47.7) | 20 (51.2) | 126 (47.2) | 0.633 |
| Hemoptysis | 98 (32.0) | 13 (33.3) | 85 (31.8) | 0.851 |
| Fever | 188 (61.4) | 24 (61.5) | 164 (61.4) | 0.989 |
| Unexplained weight loss | 237 (77.5) | 33 (84.6) | 204 (76.4) | 0.252 |
| Loss of appetite | 171 (55.9) | 22 (56.4) | 149 (55.8) | 0.943 |
| Shortness of breath | 194 (63.4) | 25 (64.1) | 169 (63.3) | 0.922 |
| Chest pain | 126 (41.2) | 12 (30.8) | 114 (42.7) | 0.157 |
| Night sweating | 167 (54.6) | 23 (58.9) | 144 (53.9) | 0.555 |
| Fatigue | 218 (71.2) | 28 (71.8) | 190 (71.2) | 0.935 |
| Presence of cavity | 137 (44.8) | 24 (61.5) | 113 (42.3) | 0.024 |
| TB treatment outcome |  |  |  |  |
| Cured or completed | 159 (51.9) | 18 (46.2) | 141 (52.8) | 0.437 |
| Loss to follow up | 33 (10.8) | 7 (17.9) | 26 (9.7) | 0.161 |
| Failed | 9 (2.9) | 0 (0.0) | 9 (3.4) | 0.610 |
| Died | 47 (15.4) | 9 (23.1) | 38 (14.2) | 0.152 |
| Not evaluated or not available | 58 (19.0) | 5 (12.8) | 53 (19.9) | 0.295 |

*IgG: immunoglobulin G, BMI: body mass index, HIV: human immunodeficiency virus, TB: tuberculosis, DS: drug-sensitive, DR: drug-resistant*

**HIV testing was conducted for 285 participant*
