# Supplementary material for: The seroprevalence of anti-Histoplasma capsulatum IgG antibody among pulmonary tuberculosis patients in seven referral tuberculosis hospitals in Indonesia
Source: PLoS Negl Trop Dis. 2023 Sep 20;17(9):e0011575. doi: 10.1371/journal.pntd.0011575 (PMC10511117; doi:10.1371/journal.pntd.0011575)
Supplement: S2 Table — (DOCX) [file pntd.0011575.s002.docx]

**S2 Table.** Details of 39 positive anti-*H. capsulatum* IgG antibody – pulmonary TB patients (1)

| **Case** | **Hospital** | **Age (year)** | **Gender** | **BMI (kg/m^2^)** | **Haemoglobin (g/dL)** | **Leukocyte (x 10^9^/L)** | **Platelet (x 10^9^/L)** | **HbA1C (%)** | **Comorbidity** | **TB treatment history** |
| --- | --- | --- | --- | --- | --- | --- | --- | --- | --- | --- |
| #01 | Sanglah, Denpasar | 51 | Female | 19.17 | 13.7 | 14.79 | 307 | 9.3 | Diabetes, Ca Ovarium | New case |
| #02 | Sanglah, Denpasar | 28 | Female | 17.26 | 11.9 | NA | NA | 5.7 | none | New case |
| #03 | Sanglah, Denpasar | 37 | Male | 24.68 | 15.8 | 4.63 | 211.7 | 5.4 | HIV-positive | New case |
| #04 | Kariadi, Semarang | 41 | Female | 14.15 | 12 | 12.6 | 401 | 8.1 | Diabetes | Past-TB history |
| #05 | Kariadi, Semarang | 32 | Female | 16.00 | 10.8 | 22.5 | 663 | 6.1 | none | Past-TB history |
| #06 | Kariadi, Semarang | 28 | Male | 14.86 | 10.2 | 14.1 | 533 | 5.7 | none | Past-TB history |
| #07 | Kariadi, Semarang | 49 | Male | 26.85 | 12.1 | 9.48 | 389 | 9.2 | Diabetes | Past-TB history |
| #08 | Kariadi, Semarang | 33 | Female | 13.42 | 9.5 | 9.6 | 420 | 5.8 | none | Past-TB history |
| #09 | Kariadi, Semarang | 54 | Male | 18.75 | 14.6 | 6.7 | 112 | 9.7 | Diabetes | Past-TB history |
| #10 | Kariadi, Semarang | 37 | Male | 22.95 | 15.2 | 8.7 | 394 | 10.6 | Diabetes | New case |
| #11 | Kariadi, Semarang | 31 | Female | 14.27 | 11.1 | 5.3 | 333 | 5.3 | none | Past-TB history |
| #12 | Kariadi, Semarang | 26 | Male | 17.58 | 13.6 | NA | NA | 5.7 | none | Past-TB history |
| #13 | Kariadi, Semarang | 46 | Male | 13.22 | 15.5 | 7.5 | 215 | 5.4 | none | New case |
| #14 | Soetomo, Surabaya | 50 | Male | 18.34 | 11.2 | 9.04 | 525 | 6.4 | none | Past-TB history |
| #15 | Soetomo, Surabaya | 45 | Female | 14.86 | 13.1 | 8.21 | 352 | 5.5 | none | Past-TB history |
| #16 | Soetomo, Surabaya | 29 | Male | 15.17 | 15.2 | 10.81 | 500 | 5.7 | none | Past-TB history |
| #17 | Soetomo, Surabaya | 52 | Male | 28.65 | 18.1 | 10.34 | 370 | 10 | Diabetes | New case |
| #18 | Soetomo, Surabaya | 44 | Female | 12.89 | 10.1 | 15.51 | 333 | 6.2 | none | Past-TB history |
| #19 | Soetomo, Surabaya | 23 | Male | 16.73 | 12.6 | 11 | 480 | 5.9 | none | New case |
| #20 | Soetomo, Surabaya | 41 | Male | 18.60 | 13.1 | 9.25 | 260 | 5.5 | none | Past-TB history |
| #21 | Soetomo, Surabaya | 45 | Female | 12.89 | 7.2 | 12.35 | 459 | 5.3 | none | New case |
| #22 | Soetomo, Surabaya | 43 | Female | 17.33 | 9.5 | 12.67 | 755 | 6.2 | none | Past-TB history |
| #23 | Soetomo, Surabaya | 62 | Male | 21.95 | 13.1 | 7.31 | 492 | 10 | Diabetes | New case |
| #24 | Soetomo, Surabaya | 31 | Male | 17.53 | 15.3 | 7.98 | 307 | 5.6 | none | Past-TB history |
| #25 | Sardjito, Yogyakarta | 63 | Male | 19.47 | 8 | 14.65 | 11 | 8 | Diabetes, AML | New case |
| #26 | Sardjito, Yogyakarta | 76 | Male | 13.05 | 11.7 | 24.49 | 1057 | 5.6 | none | New case |
| #27 | Sardjito, Yogyakarta | 54 | Female | 14.61 | 14.9 | 5.14 | 297 | 5.9 | none | Past-TB history |
| #28 | Sardjito, Yogyakarta | 64 | Female | 23.44 | 7.9 | 13.38 | 6 | 6.1 | ALL | New case |
| #29 | Persahabatan, Jakarta | 21 | Female | 23.23 | 10 | 9.06 | 435 | 5 | none | New case |
| #30 | Persahabatan, Jakarta | 62 | Male | 30.85 | 11.2 | 10.04 | 460 | 9.6 | Diabetes | New case |
| #31 | Persahabatan, Jakarta | 41 | Male | 19.47 | 11.8 | 16.65 | 609 | 10.6 | Diabetes | Past-TB history |
| #32 | Persahabatan, Jakarta | 19 | Male | 15.63 | 8.6 | 6.92 | 241 | 4.7 | none | Past-TB history |
| #33 | Persahabatan, Jakarta | 55 | Male | 17.58 | 9.9 | 13.71 | 690 | 7.6 | Diabetes | New case |
| #34 | Persahabatan, Jakarta | 57 | Male | 23.18 | 14.2 | 12.71 | 388 | 14.4 | Diabetes | Past-TB history |
| #35 | Persahabatan, Jakarta | 49 | Male | 21.88 | 12.4 | 7.51 | 252 | 13.4 | Diabetes | New case |
| #36 | Persahabatan, Jakarta | 58 | Male | 22.49 | 13.6 | 10.15 | 343 | 8.6 | Diabetes | New case |
| #37 | Persahabatan, Jakarta | 56 | Male | 14.84 | 10.7 | 9.22 | 288 | 5.5 | none | New case |
| #38 | Persahabatan, Jakarta | 47 | Male | 15.57 | 13.9 | 8.84 | 342 | 11.5 | Diabetes | Past-TB history |
| #39 | Adam Malik, Medan | 56 | Male | 21.97 | 13.6 | 14.06 | 359 | 12.3 | Diabetes | Past-TB history |

*AML: acute myeloid leukemia, ALL: acute lymphocytic leukemia, Ca: carcinoma, HIV: human immunodeficiency virus, NA: not available, TB: tuberculosis*

**S2 Table.** Details of 39 positive anti-*H. capsulatum* IgG antibody – pulmonary TB patients (2)

| **Case** | **Productive Cough** | **Haemoptysis** | **Fever** | **Weight loss** | **Loss of appetite** | **Shortness of breath** | **Chest pain** | **Night sweat** | **Fatigue** | **Cavity in Chest X-Ray** | **TB Category** | ***H. capsulatum* IgG Antibody Unit Value** | **TB Treatment Outcome** |
| --- | --- | --- | --- | --- | --- | --- | --- | --- | --- | --- | --- | --- | --- |
| #01 | **✔** | - | - | **✔** | - | **✔** | - | - | - | Upper | DS-TB | 26.45 | Cured |
| #02 | **✔** | - | **✔** | - | - | - | - | - | **✔** | Middle | DR-TB | 9.05 | Cured |
| #03 | **✔** | **✔** | **✔** | - | - | - | **✔** | - | - | Lower | Clinical-TB | 47.97 | Not available |
| #04 | **✔** | - | **✔** | **✔** | **✔** | **✔** | - | - | **✔** | Upper, middle, & lower | DR-TB | 9.35 | Cured |
| #05 | - | - | **✔** | **✔** | **✔** | **✔** | - | **✔** | - | Upper & middle | DR-TB | 12.36 | Cured |
| #06 | **✔** | - | - | **✔** | - | **✔** | - | - | **✔** | Upper & middle | DR-TB | 10.27 | Died |
| #07 | **✔** | - | **✔** | **✔** | - | **✔** | - | **✔** | - | Middle | DR-TB | 10.44 | Died |
| #08 | **✔** | - | **✔** | **✔** | **✔** | **✔** | - | - | **✔** | Upper | DR-TB | 24.92 | Died |
| #09 | **✔** | - | **✔** | - | - | **✔** | - | **✔** | **✔** | Upper, middle, & lower | DR-TB | 8.75 | Died |
| #10 | **✔** | **✔** | **✔** | **✔** | - | - | - | - | **✔** | Upper & middle | DR-TB | 8.64 | Cured |
| #11 | **✔** | - | **✔** | **✔** | - | **✔** | - | - | **✔** | Upper, middle, & lower | DR-TB | 14.35 | Died |
| #12 | **✔** | **✔** | **✔** | **✔** | **✔** | **✔** | - | - | - | - | DR-TB | 13.22 | Cured |
| #13 | **✔** | - | **✔** | **✔** | **✔** | **✔** | **✔** | **✔** | **✔** | Upper | DR-TB | 15.72 | Cured |
| #14 | **✔** | - | - | **✔** | - | **✔** | - | **✔** | - | Upper & middle | DS-TB | 25.89 | Cured |
| #15 | **✔** | - | **✔** | **✔** | **✔** | **✔** | **✔** | **✔** | **✔** | Upper & middle | DS-TB | 8.77 | Cured |
| #16 | **✔** | - | - | **✔** | **✔** | **✔** | - | **✔** | **✔** | - | DR-TB | 9.15 | Lost to follow up |
| #17 | **✔** | **✔** | - | **✔** | - | **✔** | - | - | **✔** | Upper | DR-TB | 11.29 | Lost to follow up |
| #18 | **✔** | - | **✔** | **✔** | **✔** | **✔** | - | - | **✔** | Upper | DR-TB | 46.12 | Cured |
| #19 | **✔** | **✔** | **✔** | **✔** | - | **✔** | - | **✔** | **✔** | - | DS-TB | 9.10 | Cured |
| #20 | **✔** | **✔** | **✔** | **✔** | **✔** | - | - | **✔** | - | - | Clinical-TB | 22.98 | Withdrew |
| #21 | **✔** | - | **✔** | **✔** | **✔** | - | **✔** | - | **✔** | - | DS-TB | 19.68 | Cured |
| #22 | **✔** | - | **✔** | **✔** | **✔** | **✔** | **✔** | **✔** | **✔** | Lower | DR-TB | 40.91 | Cured |
| #23 | **✔** | **✔** | **✔** | **✔** | **✔** | **✔** | **✔** | **✔** | **✔** | - | DR-TB | 8.79 | Lost to follow up |
| #24 | **✔** | - | **✔** | - | - | - | **✔** | - | **✔** | - | DR-TB | 10.11 | Lost to follow up |
| #25 | **✔** | **✔** | **✔** | **✔** | **✔** | - | - | **✔** | - | - | Clinical-TB | 33.44 | Died |
| #26 | **✔** | - | - | **✔** | **✔** | **✔** | - | **✔** | **✔** | - | DS-TB | 12.05 | Died |
| #27 | **✔** | **✔** | - | **✔** | **✔** | - | - | **✔** | - | - | Clinical-TB | 16.61 | Cured |
| #28 | **✔** | - | **✔** | - | **✔** | - | - | - | **✔** | - | Clinical-TB | 49.30 | Died |
| #29 | **✔** | **✔** | - | **✔** | - | **✔** | **✔** | **✔** | **✔** | Upper | DS-TB | 20.69 | Lost to follow up |
| #30 | **✔** | - | - | **✔** | **✔** | **✔** | - | **✔** | **✔** | - | Clinical-TB | 42.68 | Not available |
| #31 | **✔** | - | **✔** | **✔** | **✔** | **✔** | **✔** | **✔** | **✔** | - | DS-TB | 15.87 | Died |
| #32 | **✔** | - | - | **✔** | **✔** | **✔** | **✔** | **✔** | **✔** | Upper & middle | DS-TB | 14.93 | Not available |
| #33 | **✔** | - | - | **✔** | - | - | **✔** | **✔** | **✔** | Upper & middle | DR-TB | 20.11 | Cured |
| #34 | **✔** | **✔** | - | **✔** | - | - | - | - | **✔** | Upper | DS-TB | 31.32 | Lost to follow up |
| #35 | **✔** | - | - | **✔** | - | - | - | **✔** | - | Upper | DS-TB | 45.02 | Cured |
| #36 | **✔** | **✔** | - | **✔** | **✔** | - | - | - | **✔** | Upper | DS-TB | 9.83 | Completed |
| #37 | **✔** | - | - | **✔** | - | **✔** | - | **✔** | **✔** | Middle | DS-TB | 12.59 | Lost to follow up |
| #38 | **✔** | **✔** | **✔** | **✔** | **✔** | - | - | **✔** | **✔** | - | DS-TB | 35.79 | Completed |
| #39 | **✔** | - | **✔** | - | **✔** | **✔** | **✔** | **✔** | - | - | DS-TB | 33.16 | Withdrew |

*DS: drug-sensitive, DR: drug-resistant, IgG: immunoglobulin G*
