# Supplementary material for: The seroprevalence of anti-Histoplasma capsulatum IgG antibody among pulmonary tuberculosis patients in seven referral tuberculosis hospitals in Indonesia
Source: PLoS Negl Trop Dis. 2023 Sep 20;17(9):e0011575. doi: 10.1371/journal.pntd.0011575 (PMC10511117; doi:10.1371/journal.pntd.0011575)
Supplement: Attachment — Submitted filename: Letter_Histoplasmosis Response to Reviewers rev - edit.docx [file pntd.0011575.s004.docx]

Jakarta, July 2023

Dear Editors and Reviewers of PLOS Neglected Tropical Diseases,

We would like to thank the editors and reviewers for the constructive and helpful comments and suggestions. We have addressed the queries and revised the manuscript for consideration by PLOS Neglected Tropical Diseases.

We have uploaded the following:

1. A letter containing a detailed list of our responses to the review comments and a description of the changes we have made in the manuscript.
2. Two versions of the revised manuscript: one with yellow highlights denoting where the text has been changed and the clean version.

Thank you very much.

Best regards,

Retno Wahyuningsih

We have carefully considered the reviewer’s comments and provide itemized responses. We also provide the author summary section in this new version of our manuscript.

**Lines 64-83** (clean version) now read:

**Author Summary**

*Histoplasma capsulatum* exposure is rarely suspected in Indonesia, even though this fungal presence in the country has been reported since the 1930s. Pulmonary manifestations of histoplasmosis, especially chronic pulmonary histoplasmosis (CPH), resemble pulmonary tuberculosis (TB). It may be misdiagnosed as or be a co-infection with pulmonary TB. Since Indonesia is the second-highest TB-burdened country worldwide, it is imperative to capture histoplasmosis data among pulmonary TB patients. Our study provides evidence of *H. capsulatum* exposure by measuring anti-*H. capsulatum* IgG antibodies in both bacteriologically confirmed and clinically-diagnosed pulmonary TB patients at seven TB referral hospitals in Indonesia. We found 12.7% (39/306) of pulmonary TB patients were positive for anti-*H. capsulatum* IgG antibodies. We had a high suspicion of pulmonary histoplasmosis diagnosis in 10 of the 39 patients (six bacteriologically confirmed and four clinically-diagnosed TB) who were immunocompromised due to high IgG assay values (>30 EU). The utilization of other modalities, such as the *Histoplasma* antigen test, may support a histoplasmosis diagnosis. In our study, malignancies and cavitary lesions were associated with anti-*H. capsulatum* IgG antibody positive results. Our results bring much-needed preliminary data to guide policymakers regarding fungal infection in pulmonary TB patients and support further research on histoplasmosis in Indonesia.

**Responses to Reviewers**

**Reviewer #1:**

This is a nice study from the team from Indonesia. The group found a relatively high prevalence of anti-histoplasma antibody titres in some patients. However, the manuscript doesn't answer the question: "Who should be screened?" I would suggest that the authors run inferential statistics to answer the above questions, which is of epidemiologic significance. There are so many people with TB, histoplasmosis is rare, so who should we screen?”

**Response:** Thank you for your invaluable input. We performed inferential statistics and modified the manuscript thoroughly to improve the epidemiological significance. We used Pearson Chi-square or Fisher’s exact test to assess differences in baseline characteristics between positive and negative anti-*H. capsulatum* IgG antibody samples. Bivariate logistic regression was used to assess factors associated with anti-*H. capsulatum* IgG antibody positive results. All independent variables with p-value <0.25 were included in multivariate logistic regression models and adjusted odds ratios (aORs) with 95% CIs were used to determine the strength of association between the dependent and independent variables. A p-value (two-tailed) <0.05 was considered statistically significant. **We found that malignancies and cavitary lesions in the lung were significantly associated with the anti-*H. capsulatum* IgG antibody positive result with OR 4.88, 95% CI 1.09–21.69 and OR 2.27, 95% CI 1.09–4.70, respectively. Therefore, screening for histoplasmosis in pulmonary TB patients who have malignancies or cavitary lesions in the lung may be beneficial.**

Details of additional changes related to these inferential statistics are below (line numbers are referred to clean version of the manuscript):

**Abstract**

**Methodology**

**Lines 42-44** now read:

…. Bivariate and multivariate logistic regression analysis were performed to assess factors associated with anti-*H. capsulatum* IgG antibody positive result.

**Results**

**Lines 52-54** now read:

…. . Factors associated with the anti-*H. capsulatum* IgG antibody positive result were malignancies (OR 4.88, 95% CI 1.09–21.69, p = 0.037) and cavitary lesions in the lung (OR 2.27, 95% CI 1.09–4.70, p = 0.028).

**Methods**

**Data and statistical methods**

**Lines 190-198** now read:

…. Differences in baseline characteristics between positive and negative anti-*H. capsulatum* IgG antibody samples were evaluated using Pearson Chi-square or Fisher’s exact test, as appropriate. Bivariate logistic regression was used to assess factors associated with anti-*H. capsulatum* IgG antibody positive result. All independent variables with p-value <0.25 were included in multivariate logistic regression models and adjusted odds ratios (aORs) with 95% CIs were used to determine the strength of association between the dependent and independent variables. A p-value (two-tailed) <0.05 was considered statistically significant. Statistical Package for Social Science (SPSS) software version 23 (IBM Corporation, Armonk, NY, USA) and GraphPad Prism 9.0 (GraphPad Software, Inc., California, USA) were used for the statistical analysis and graphs.

**Results**

**Lines 256-265** now read:

Table 2. presents factors associated with the anti-*H. capsulatum* IgG antibody positive result. Cavitary lesions in the lung were significantly associated with the anti-*H. capsulatum* IgG antibody positive result as evaluated by bivariate analysis. The multivariate logistic regression analysis included the variables with a p-value of <0.25. Factors independently associated with the anti-*H. capsulatum* IgG antibody positive result were malignancies (OR 4.88, 95% CI 1.09–21.69, p = 0.037) and cavitary lesions in the lung (OR 2.27, 95% CI 1.09–4.70, p = 0.028).

Table 2. Bivariate and multivariate logistic regression analyses of factors associated with the anti-*H. capsulatum* IgG antibody positive result.

|  | **Positive anti-*H. capsulatum* IgG**  **No. (%)**  **N = 39** | **Negative anti-*H. capsulatum* IgG**  **No. (%)**  **N = 267** | **Bivariate analysis** | | **Multivariate analysis** | |
| --- | --- | --- | --- | --- | --- | --- |
|  |  |  | **OR**  **(95% CI)** | **p-value** | **Adjusted OR**  **(95% CI)** | **p-value** |
| Gender |  |  |  |  |  |  |
| Male | 26 (66.7) | 154 (57.7) | 1.47  (0.72-2.98) | 0.289 |  |  |
| Female | 13 (33.3) | 113 (42.3) |  |  |  |  |
| Age (years) |  |  |  |  |  |  |
| 18-44 | 18 (46.2%) | 147 (55.1%) | *Ref.* |  |  |  |
| 45-59 | 16 (41.0%) | 92 (34.5%) | 1.42  (0.69-2.92) | 0.341 |  |  |
| ≥60 | 5 (12.8%) | 28 (10.5%) | 1.46  (0.50-4.25) | 0.490 |  |  |
| Site location with available histoplasmosis surveillance data |  |  | 1.46  (0.73-2.92) | 0.289 |  |  |
| Yes (Bali, Surabaya, Jakarta, and Medan) | 25 (64.1) | 147 (55.1) |  |  |  |  |
| No (Semarang, Yogyakarta, and Makassar) | 14 (35.9) | 120 (44.9) |  |  |  |  |
| BMI (kg/m^2^) |  |  |  |  |  |  |
| < 18.5 (underweight) | 22 (56.4) | 133 (49.8) | 1.30  (0.66-2.57) | 0.442 |  |  |
| ≥18.5 | 17 (43.6) | 134 (50.2) |  |  |  |  |
| Previous TB treatment history |  |  |  |  |  |  |
| Yes | 21 (53.8) | 111 (41.6) | **1.64**  **(0.84-3.22)** | **0.151** | 1.73  (0.84-3.55) | 0.135 |
| No | 18 (46.2) | 156 (58.4) |  |  |  |  |
| Anemia |  |  |  |  |  |  |
| Yes | 27 (69.2) | 170 (63.7) | 1.28  (0.62-2.65) | 0.499 |  |  |
| No | 12 (30.8) | 97 (36.3) |  |  |  |  |
| Positive HIV (available data in 285 participants) |  |  |  |  |  |  |
| Yes | 1 (2.6) | 16 (5.9) | 0.43  (0.06-3.35) | 0.420 |  |  |
| No | 34 (97.1) | 234 (93.6) |  |  |  |  |
| Diabetes |  |  |  |  |  |  |
| Yes | 16 (41.0) | 92 (34.5) | 1.32  (0.67-2.63) | 0.424 |  |  |
| No | 23 (59.0) | 175 (65.5) |  |  |  |  |
| Malignancies |  |  |  |  |  |  |
| Yes | 3 (7.7) | 7 (2.6) | **3.09**  **(0.77-12.51)** | **0.113** | **4.88**  **(1.09-21.69)** | **0.037** |
| No | 36 (92.3) | 260 (97.4) |  |  |  |  |
| Unexplained weight loss |  |  |  |  |  |  |
| Yes | 33 (84.6) | 204 (76.4) | 1.69  (0.68-4.24) | 0.256 |  |  |
| No | 6 (15.4) | 63 (23.6) |  |  |  |  |
| Hemoptysis |  |  |  |  |  |  |
| Yes | 13 (33.3) | 85 (31.8) | 1.07  (0.52-2.19) | 0.851 |  |  |
| No | 26 (66.7) | 182 (68.2) |  |  |  |  |
| Fever |  |  |  |  |  |  |
| Yes | 24 (61.5) | 164 (61.4) | 1.01  (0.50-2.01) | 0.989 |  |  |
| No | 15 (38.5) | 103 (38.6) |  |  |  |  |
| Shortness of breath |  |  |  |  |  |  |
| Yes | 25 (64.1) | 169 (63.3) | 1.04  (0.51-2.09) | 0.922 |  |  |
| No | 14 (35.9) | 98 (36.7) |  |  |  |  |
| Chest pain |  |  |  |  |  |  |
| Yes | 12 (30.8) | 114 (42.7) | **0.59**  **(0.29-1.23)** | **0.161** | 0.57  (0.27-1.21) | 0.143 |
| No | 27 (69.2) | 153 (57.3) |  |  |  |  |
| Presence of cavity |  |  |  |  |  |  |
| Yes | 24 (61.5) | 113 (42.3) | **2.18**  **(1.09-4.34)** | **0.027** | **2.27**  **(1.09-4.70)** | **0.028** |
| No | 15 (38.5) | 154 (57.7) |  |  |  |  |
| TB category |  |  |  |  |  |  |
| DS-TB | 15 (38.5) | 100 (37.5) | *Ref.* |  |  |  |
| DR-TB | 18 (46.2) | 96 (35.9) | 1.25  (0.59-2.62) | 0.555 |  |  |
| Clinically-diagnosed TB | 6 (15.4) | 71 (26.6) | 0.563  (0.21-1.52) | 0.258 |  |  |

**Discussion**

**Lines 379-389** now read:

…. Our study found that pulmonary TB patients with malignancies or cavitary lesions in the lung were significantly associated with the anti-*H. capsulatum* IgG antibody positive result. Previous studies have described the association between pulmonary cavitation and immunocompromised status with fungal co-infection in pulmonary TB patients [42]. Cavities resemble an ideal culture plate for fungal colonization [42]. Patients with malignancies are exposed to long-term chemotherapy or immunosuppressant that decreases immunity, thus making the patients more susceptible to fungal co-infection [42]. Screening for histoplasmosis may be beneficial in pulmonary TB patients with malignancies or cavitary lesions, both bacteriologically TB-confirmed and clinically-diagnosed TB. However, our findings should be interpreted cautiously because the positive results of the IgG antibody may reflect past exposure.

**Conclusions**

**Lines 413-415** now read:

….. Pulmonary TB patients, both bacteriologically TB-confirmed and clinically-diagnosed TB, with malignancies or cavitary lesions in the lung were associated with the anti-*H. capsulatum* IgG antibody positive result in our study.

**Reviewer #2**

*Methods*

1. The population is clearly described and appropriate though it would have been interesting to have a group of asymptomatic participants.

**Response:** Thank you for your comment. This work was a sub-study of a previous pulmonary TB study (TRIPOD). The TRIPOD subject is symptomatic pulmonary TB patients (bacteriologically TB confirmed and clinically-diagnosed TB) and we followed them during TB treatment. Therefore, we did not have a group of asymptomatic participants. We agree that it will be interesting to perform histoplasmosis testing in asymptomatic participants to assess endemicity status and analyze the differences with symptomatic patients. We will highly consider it for further histoplasmosis studies in Indonesia.

2. Correct statistical analysis was used. But testing for possible associated risk factors may add more value to the manuscript.

**Response:** Thank you for highlighting this important point. The other reviewer also suggested performing inferential statistics to improve the epidemiological significance. We performed Pearson Chi-square or Fisher’s exact test to assess differences in baseline characteristics between positive and negative anti-*H. capsulatum* IgG antibody samples. Bivariate logistic regression was used to assess factors associated with anti-*H. capsulatum* IgG antibody positive result. All independent variables with p-value <0.25 were included in multivariate logistic regression models and adjusted odds ratios (aORs) with 95% CIs were used to determine the strength of association between the dependent and independent variables. A p-value (two-tailed) <0.05 was considered statistically significant. **We found that malignancies and cavitary lesions in the lung were significantly associated with the anti-*H. capsulatum* IgG antibody positive result with OR 4.88, 95% CI 1.09–21.69 and OR 2.27, 95% CI 1.09–4.70, respectively. Therefore, screening for histoplasmosis in pulmonary TB patients who have malignancies or cavitary lesions in the lung may be beneficial.** We have modified the current manuscript to accommodate these changes. Please refer to responses to Reviewer 1.

The work is of health importance. However, some worries need to be addressed:

1. Why was Histoplasma antigen not also tested here as it may have help in disease classification?

**Response:** Thank you, we appreciate your concern. This is a very important point. We agree that utilization of *Histoplasma* antigen test may support a histoplasmosis diagnosis. We emphasized it in the discussion section (Line 353-354): *In a clinical setting, clinicians should combine antibody testing with antigen detection assays to verify a histoplasmosis diagnosis*. However, our study was designed to evaluate the seroprevalence of anti-*H. capsulatum* IgG antibody. Therefore, the study funding was only available for purchasing and importing the IgG antibody tests; thus, the antigen test was not provided. Also, the antigen test is more useful in establishing disseminated histoplasmosis, which is usually uncommon in pulmonary TB patients.

We now add this limitation to the study limitation section.

**Lines 402-406** now read:

*Histoplasma* antigen test is feasible in resource-limited settings to support histoplasmosis diagnosis. However, our study was designed to evaluate the seroprevalence of anti-*H. capsulatum* IgG antibody; thus, the antigen test was not provided. Also, the antigen test is more useful in establishing disseminated histoplasmosis, which is usually uncommon in pulmonary TB patients.

2. I expect the study population to be "presumptive pulmonary TB patients" and not pulmonary TB patients as one of your study group is made of not microbiological confirmed PTB patients. please modify all over the manuscript.

**Response:** Thank you for your suggestions. The study population was presumptive pulmonary TB adult patients. After microbiological TB testing (acid-fast bacilli (AFB) smear, Xpert MTB/RIF, and culture), the clinician decided on the pulmonary TB diagnosis. Participants were terminated from the study if they were not diagnosed and treated as pulmonary TB patients. Therefore, for our study, we analyzed pulmonary TB patients (both pulmonary TB bacteriologically confirmed and clinically-diagnosed TB without microbiological evidence of TB). We clarified the study group definition in the abstract and method section.

**Abstract**

**Methodology**

**Lines 38-40** now read:

The study population was presumptive pulmonary TB adult patients who underwent microbiological TB examinations and were categorized as drug-sensitive (DS), drug-resistant (DR), and clinically-diagnosed TB.

**Methods**

**Lines 151-163** now read:

The study population was presumptive pulmonary TB adult patients (with or without a history of anti-TB treatment) attended to the hospital with a cough for at least two weeks and at least one other TB symptom (fever, unexplained weight loss, loss of appetite, hemoptysis, shortness of breath, chest pain, night sweats, or fatigue) and chest X-ray suggestive TB. The TRIPOD study successfully enrolled 490 participants and collected sputum for microbiological TB testing (acid-fast bacilli (AFB) smear, Xpert MTB/RIF, and *Mycobacterium tuberculosis* (MTB) culture) and blood for laboratory testing. Participants were terminated from the study if they were not treated as pulmonary TB patients by the clinician. Participants who were treated as pulmonary TB patients, according to microbiological TB and drug susceptibility tests were further categorized as pulmonary TB bacteriologically confirmed, with sub-categorization as drug-sensitive (DS-TB) or drug-resistant (DR-TB), and clinically-diagnosed TB when there was no microbiological evidence of TB.

3. Line 75: From " *Other immunocompromised states risk severe histoplasmosis, including solid organ transplantation, chronic inflammatory diseases, autoimmune diseases, innate immunodeficiencies, inborn T-cell immunodeficiencies, poorly controlled diabetes, and hematologic malignancies*", please rephrase for clarity.

**Response:** Thank you for your suggestions, we rephrased the sentence for clarity.

**Lines 94-98** now read:

…. Several immunocompromised conditions aside from HIV infection also increase the likelihood of severe histoplasmosis, including solid organ transplantation, chronic inflammatory diseases, autoimmune diseases, innate immunodeficiencies, inborn T-cell immunodeficiencies, poorly controlled diabetes, and hematologic malignancies.

4. Line 104: “*Furthermore, TB and pulmonary histoplasmosis can coincide, especially in high TB burden countries such as India, China, and Africa*”, use another word to replace countries. You may use "setting", "area" ...or remove Africa and cite instead some African countries.

**Response:** Thank you for your suggestions, we removed “countries” and cited some African countries according to references.

**Lines 123-124** now read:

…, especially in high TB burden areas such as India, China, Nigeria, and South Africa.

5. Line 224: from "*Our finding that 12.7% of tested TB patients had anti-H. capsulatum IgG antibody aligns with previous surveillance reports of different populations from the areas of Jakarta, Surabaya, Kedisan, and Medan, Indonesia*", please rephrase for better understanding.

**Response:** Thank you for your suggestions, we rephrased the sentence for better understanding.

**Lines 268-271** now read:

…. Anti-*H. capsulatum* IgG antibody was detected in 12.7% (39/306) of our study population, consistent with previous surveillance reports conducted in several populations in Indonesia such as Jakarta, Surabaya, Kedisan, and Medan.
